# Left ventricular T2 distribution in Duchenne Muscular Dystrophy

**DOI:** 10.1186/1532-429X-12-14

**Published:** 2010-03-18

**Authors:** Janaka P Wansapura, Kan N Hor, Wojciech Mazur, Robert Fleck, Sean Hagenbuch, D Woodrow Benson, William M Gottliebson

**Affiliations:** 1 Division of Radiology, Cincinnati Children's Hospital Medical Center, Cincinnati, Ohio, USA; 2 Division of Cardiology, Cincinnati Children's Hospital Medical Center, Cincinnati, Ohio, USA; 3 Division of Cardiology, The Ohio Heart and Vascular Center, Christ Hospital, Cincinnati, Ohio, USA

## Abstract

**Background:**

Although previous studies have helped define the natural history of Duchenne Muscular Dystrophy (DMD)-associated cardiomyopathy, the myocardial pathobiology associated with functional impairment in DMD is not yet known.

The objective of this study was to assess the distribution of transverse relaxation time (T2) in the left ventricle (LV) of DMD patients, and to determine the association of myocardial T2 heterogeneity to the severity of cardiac dysfunction. DMD patients (n = 26) and normal control subjects (n = 13) were studied by Cardiovascular Magnetic Resonance (CMR). DMD subject data was stratified based on subject age and LV Ejection Fraction (EF) into the following groups: A (<12 years old, n = 12); B (≥12 years old, EF ≤ 55%, n = 8) and C (≥12 years old, EF = 55%, n = 6). Controls were also stratified by age into Groups N1 (<12 years, n = 6) and N2 (>12 years, n = 5). LV mid-slice circumferential myocardial strain (ε_cc_) was calculated using tagged CMR imaging. T2 maps of the LV were generated for all subjects using a black blood dual spin echo method at two echo times. The Full Width at Half Maximum (*FWHM*) was calculated from a histogram of LV T2 distribution constructed for each subject.

**Results:**

In DMD subject groups, *FWHM *of the T2 histogram rose progressively with age and decreasing EF (Group A *FWHM*= 25.3 ± 3.8 ms; Group B *FWHM*= 30.9 ± 5.3 ms; Group C *FWHM*= 33.0 ± 6.4 ms). Further, *FWHM *was significantly higher in those with reduced circumferential strain (|ε_cc_| ≤ 12%) (Group B, and C) than those with |ε_cc_| > 12% (Group A). Group A *FWHM *was not different from the two normal groups (N1 *FWHM *= 25.3 ± 3.5 ms; N2 *FWHM*= 24.0 ± 7.3 ms).

**Conclusion:**

Reduced EF and ε_cc _correlates well with increased T2 heterogeneity quantified by *FWHM*, indicating that subclinical functional impairments could be associated with pre-existing abnormalities in tissue structure in young DMD patients.

## Background

Duchenne Muscular Dystrophy (DMD), a lethal X-linked skeletal and cardiac myopathy, affects 1/3500 males [1]. DMD is caused by mutations in the dystrophin gene resulting in greatly reduced or absent dystrophin protein, and is characterized by progressive skeletal muscle weakness, with loss of ambulation typically during the second decade [2,3]. A proposed mechanism of DMD pathogenesis in cardiac muscle is an alteration in calcium (Ca^2+^) homeostasis, whereby micro-tears in the sarcolemmal membrane and non-specific channel opening events initiate a destructive cascade culminating in myocyte necrosis and fibrosis [4-6]. Studies have shown that occult ventricular dysfunction and myocardial fibrosis can be diagnosed by Cardiovascular Magnetic Resonance (CMR) in DMD [7,8]. Previously, we found that peak left ventricular composite myocardial circumferential strain (ε_cc_) is reduced early in the course of DMD despite normal ejection fraction (EF), and the strain values continue to decline with advancing age[9]. While this and other studies have helped define the natural history of DMD-associated cardiac dysfunction by characterizing changes in myocardial mechanics, assessment of the myocardial pathobiology associated with the regional functional impairment in DMD has not been described. As such, an index of DMD-associated cardiac dysfunction that defines the pathobiology of the disease would enhance our ability to assess the natural history of this disease.

A CMR methodology for evaluating *in-vivo *pathobiology has, however, been available for quite some time. The measure of transverse relaxation time (T2) of tissue has been well-characterized; T2 weighted MR signal has been used to detect various pathologies of soft tissues [10,11]. Factors such as fatty infiltration [12], increased water content and vasogenic edema[13] are known to increase T2, while collagen accumulation, as seen in extracellular matrix fibrosis, is known to decrease T2 [14]. In recent years, T2 weighted imaging, as well as absolute T2 mapping, has been demonstrated to be valuable in cardiac imaging of acute myocardial process such as myocardial infarct [15-17], myocarditis [18,19], and acute cell mediated transplant rejection[20].

DMD is an example of a progressive disease process where fibrosis and edema related to inflammation coexist. In a previous study conducted at a magnetic field strength of 0.5 Tesla, Mavrogeni et al [21] showed that DMD patients had lower cardiac T2 relaxation time which progressively decreased with age compared to control subjects. The same authors recently showed that with steroid treatment, myocardial T2 increased in older DMD patients [22]. However, Mavrogeni et al [21] limited their consideration to the mean T2 value in the septal region of the heart, forgoing analysis of T2 distribution across multiple regions of the LV. However, we and others have shown [23,24] that myocardial fibrosis and regional abnormalities appear heterogeneously, with initial appearance of abnormalities typically in the LV free wall.

We thus hypothesized that heterogeneity of T2 across an entire slice of the heart exist in the early stages of DMD-associated cardiomyopathy and are associated with the subclinical functional abnormalities quantified by myocardial circumferential strain. The purpose of this study was thus to evaluate the heterogeneity of T2 distribution in the LV in boys with various stages of DMD.

## Methods

### Study population

Data were analyzed from records of DMD subjects followed at Cincinnati Children's Hospital Medical Center. The diagnosis of DMD was confirmed by skeletal muscle biopsy showing absent dystrophin and/or DNA analysis demonstrating a characteristic dystrophin mutation in all patients. DMD patients who underwent clinical CMR between September 2005 and September 2007 were included in this analysis. Additionally, two control groups of young (N1, n = 8, age 5-10 years) and older (N2, n = 5, age 32-45 years) normal control males underwent an identical protocol. EF and |ε_cc_| measurements were not available in the N2 control group. The Institutional Review Board at the Cincinnati Children's Hospital Medical Center approved the study.

All subjects were > 5 years of age, thereby eliminating the need for sedation. CMR was performed at our center on 97 DMD patients between September 2005 and September 2007. Data from 71 was excluded due to absence of T2 images (n= 44), absence of tagged images (n = 18) or poor tag quality secondary to breathing artifact or patient movement (n = 9).

### Subject stratification

**Figure 1:**
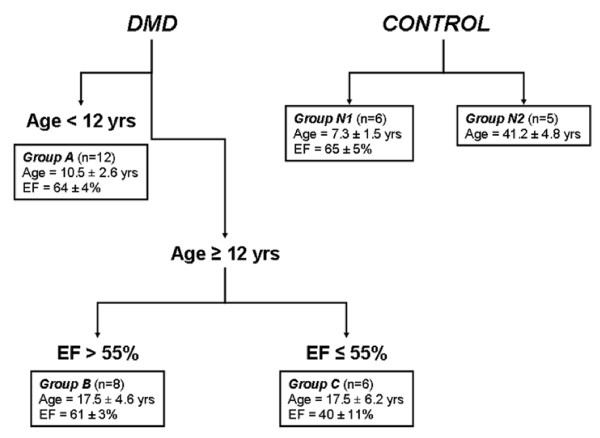
**Categorization of the study population**. Stratification of Duchenne muscular dystrophy (DMD) patients based on age and ejection fraction (EF). Normal controls were stratified according to age given their dichotomous distribution.

The subject data were stratified into 5 groups (two groups of control subjects: N1(n = 6) and N2(n = 5)) and three groups of DMD patients (A (n = 12), B (n = 8), and C (n = 6)). Stratification in the control subjects was based on age. In the DMD subjects stratification was primarily by age and secondarily by LV Ejection Fraction (EF) (Figure 1). Thus, Group A comprised DMD subjects age <12 years with normal EF. Group B were DMD subjects age ≥12 years with normal EF. Group C included DMD subjects age ≥12 years with reduced EF (≤55%). Due to the significant dichotomy in ages of the control subjects, they were also stratified by age into Groups N1 (<12 years) and N2 (>12 years), due to the significant dichotomy in ages of these subjects.

### CMR acquisition

CMR was performed on a Siemens 3-T *Trio *(Siemens Medical Solutions, Malvern, Pennsylvania/Erlangen, Germany) system.

### Imaging protocols

#### Ventricular Volumetry and Global Functional Imaging

Cardiac functional imaging was performed as previously described [9]. CMR was acquired with retrospective electrocardiogram (ECG)-gating, segmented steady-state free precession technique after localized shimming and/or frequency adjusting. Subjects were breath-held as tolerated; for those subjects who could not adequately breath-hold, a free breathing technique with multiple signal averaging was used. Standard imaging included a short-axis stack of cine SSFP images from cardiac base to apex.

#### Myocardial Strain Imaging

Tagged cine CMR were acquired in the short axis of the mid left ventricle at the level of the papillary muscles with an ECG-triggered segmented k-space fast gradient echo sequence with spatial modulation of magnetization in orthogonal planes. Grid tag spacing was 7 to 8 mm. The scan parameters used were: field of view (30 - 32) × (25 - 26) cm^2^, slice thickness 6 mm, flip angle 20°, TE/TR 3 ms/4.2 ms, views/segment 7-9.

#### T2 Imaging

For T2 measurements, spin echo images of the left ventricle in the short axis plane were acquired using a black blood dual spin echo method. Imaging parameters were: Slice thickness = 5 mm, in plane resolution = 1.4 mm × 1.4 mm, echo train length = 5, Echo times: TE_1 _= 6 ms, TE_2 _= 34 ms. Data acquisition was done during late diastole by inserting a subject dependent delay.

### Data analysis

#### Ventricular Volumetry and EF

Ventricular volumes, mass, and global function were assessed via standard planimetry techniques with semi-automated computer software (QMASS version 6.1.5, Medis Medical Imaging Systems, Leiden, the Netherlands) by expert readers (R.J.F., W.M.G., and K.N.H.).

#### Myocardial strain analysis

Tagged images were analyzed with the HARmonic Phase (HARP, Diagnosoft, Palo Alto, California) technique, as previously described [9]. Based on our experience and that of others regarding limited reproducibility of the basal and apical slices, only the midventricular slice was analyzed [8]. An average of all the regional values/subject was calculated as a composite whole-slice strain value ε_cc_, to allow for comparison purposes. All HARP strain analyses were performed by an experienced reader (K.N.H.). To assess inter-observer variability of HARP analysis, a second experienced reader (W.M.G) performed the same analysis on a subset of DMD subjects (n = 6) and controls (n = 3).

#### T2 Analysis

T2 values were calculated on a pixel-by-pixel basis according to the standard description of the signal behavior in spin echo magnetic resonance imaging as:(1)

where S_1 _and S_2 _are the signal intensity at echo times TE_1 _and TE_2 _respectively. Regions of interests were manually drawn (J.P.W.) to extract the T2 values. The LV was defined on the T2 weighted magnitude image (i.e. at TE = TE_2_) by manually drawing (J.P.W) the endo- and epicardial boarders with care taken to avoid signal from blood. Similarly, lateral wall and septum was segmented manually as defined by the American Heart Association 17-segmental model i.e. septum = regions 7 and 8; lateral wall = regions 11 and 12. Using these T2 values, a histogram of LV T2 distribution with bin size equal to 1 ms was constructed for each subject.

#### FWHM Calculation

For each histogram, the Full Width at Half Maximum (*FWHM*) was calculated after applying box car averaging. The *FWHM *was defined as the width of the histogram at half the maximum height. Additionally, the mean and the standard deviation of T2 of the entire LV as well as in the septum and in the lateral wall were also calculated. All image processing related to T2 measurements were done using custom developed software written in the Interactive Data Language (IDL, Research Systems Inc., CO, USA).

### Statistical analyses

Analyses were performed using SAS^® ^(Version 9.2 Cary, NC). The General Linear Model procedure was used to access group differences among the means for *FWHM *adjusting for age. Since age was not shown to be significant, it was removed from the model. The distribution of T2 was slightly skewed; therefore the Wilcoxon Rank sum test was used. All tests were two-sided and p-values ≤0.05 or confidence intervals that did not include zero were considered statistically significant.

## Results

A summery of results is presented in Table 1.

**Table 1:** Summary of CMR findings between groups. Group values are presented as mean ± standard deviation

Group	EF %	|Ecc| %	Age	T2 (ms)	*FWHM *(ms)
A (n = 12)	64 ± 4	14.2 ± 0.8*	10.5 ± 2.6 *	61 ± 9	25.3 ± 3.8

B (n = 8)	60 ± 3	11.0 ± 0.8 †*	17.5 ± 4.6 †*	58 ± 9	30.9 ± 5.3 †*

C (n = 6)	40 ± 11 †‡	7.7 ± 0.3 †‡*	17.5 ± 6.3 †*	63 ± 6	33.0 ± 6.4 †*

N1 (n = 6)	65 ± 5	20 ± 2.5	7.3 ± 1.6	57 ± 9	25.5 ± 3.5

N2 (n = 5)			41.2 ± 4.2	46 ± 11	24.0 ± 7.3

* The mean is statistically different to that of Group N1 with a p < 0.05

† The mean of Group A is statistically different to that of Group B or C with a p < 0.05

‡ The mean of Group B is statistically different to that of Group C with a p < 0.05

### EF and ε_cc _Data

All DMD subjects had abnormal |ε_cc_| < 16%, consistent with our prior work [9], despite normal EF in Groups A and B. As previously found, the mean |ε_cc_| was significantly different among the DMD groups and progressively decreased with age and EF. Control subjects in group N1 had normal EF and normal |ε_cc_| (>16%).

### T2 Data

**Figure 2:**
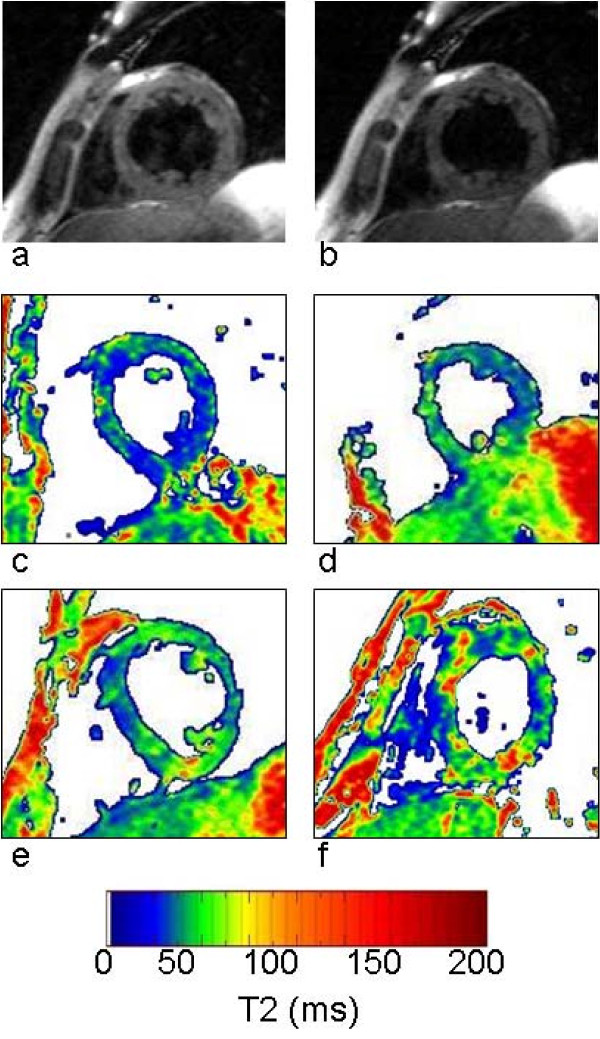
**Left Ventricular T2 Maps**. Black blood fast spin echo images of the left ventricle at TE = 6 ms (a) and TE= 34 ms (b) in the short axis view of a DMD subject. The T2 maps of a normal (c) and DMD subjects (d: group A, e: group B and f: group C) show the increasing heterogeneity of T2 in the LV corresponding to the severity of disease.

**Figure 3:**
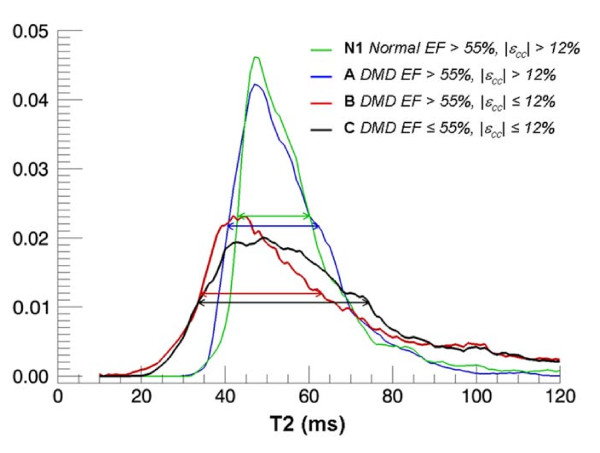
**T2 histograms**. Examples of normalized (i.e. area under curve = 1) T2 histograms of DMD (Group A, B and C) and Normal control (N1) subjects show that DMD patients with normal EF but impaired ε_cc _has higher heterogeneity in T2 compared to other groups.

**Figure 4:**
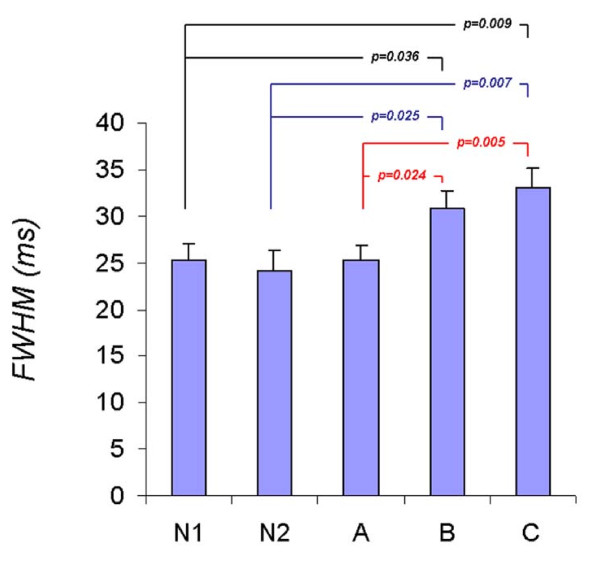
**Graphs of *FWHM*/Strata**. The *FWHM *of the DMD groups B (age >12 yrs, EF ≤ 55%, |ε_cc_| < 12%) and C (age >12 yrs EF = 55%, |ε_cc_| < 12%) were significantly higher than all other groups including group A (age <12 yrs EF > 55%, |ε_cc_| > 12%) indicating increased heterogeneity in the T2 distribution in DMD with impaired regional and global function.

Figure 2 shows the black blood T2 images acquired at echo times of 6 ms (Figure 2a) and 34 ms (Figure 2b) in a DMD subject. The T2 maps show heterogeneity of LV T2 distribution in a DMD patient (Figure 2d) compared to that of a normal subject (Figure 2c) and were quantified using T2 histograms (Figure 3). A Wilcoxon Rank Sum test showed no significant difference in the mean T2 of the entire LV among all groups. Similarly no significant difference in the standard deviation of T2 was found between groups. The age was not a significant factor in this model. However, the *FWHM *of the T2 histogram was significantly (p < 0.05) greater in Groups B (30.9 ± 5.3 ms) and C (33.0 ± 6.4 ms) compared to that of Group A (25.3 ± 3.8 ms) and the normal Groups N1 (25.3 ± 3.5 ms) and N2 (24.0 ± 7.3 ms) (Figure 4). This indicates that significantly greater T2 heterogeneity in Groups B and C with advanced cardiac dysfunction. These results also show that DMD patients with normal EF but impaired circumferential strain (|ε_cc_| ≤ 12%, Group B) had a significantly greater T2 heterogeneity than that of DMD patients with normal EF and |ε_cc_| >12% (Group A).

**Figure 5:**
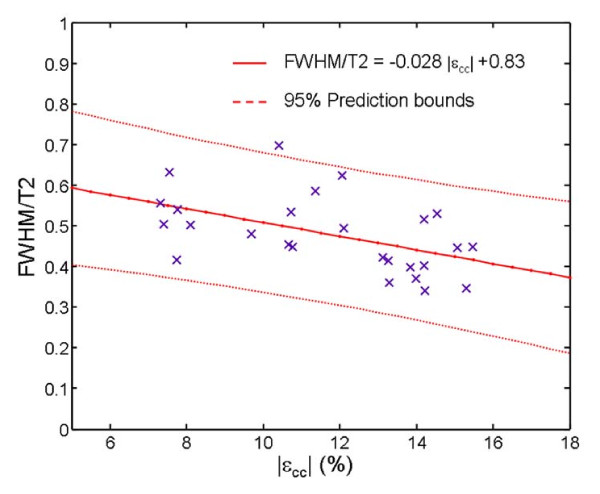
**Regression of *FWHM*/T2mean vs ε_cc_**. Regression analysis show a moderate association between *FWHM*/T2mean and ε_cc _in group A and B (Pearson correlation coefficient r = .51) suggesting the presence of microscopic fibrosis long before it becomes overt enough to be detected by the LGE technique.

Among DMD patients with Normal EF (i.e. Groups A and B), a trend towards *increasing FWHM *with *decreasing *mean T2 of the LV (T2mean) was noticed. We characterized this trend by calculating the ratio *FWHM*/T2mean for all subjects of groups A and B. *FWHM*/T2mean was significantly (p < .0001) higher in group B (0.54 ± 0.08) compared to Group A (0.42 ± 0.06). Regression analysis showed moderate association between *FWHM*/mean T2 and |ε_cc_| in group A and B (Figure 5). (Pearson correlation coefficient r = .51). Age alone was not found to be a significant factor in predicting T2 heterogeneity.

Results of regional analysis of mean T2, T2 *FWHM *and ε_cc _are shown in Table 2. Mean T1 and T2 *FWHM *were higher in the septum than in the lateral wall. No difference in the septum and lateral wall was found in ε_cc_. Although not statistically significant the lateral wall T2 *FWHM *followed the same trend as T2 *FWHM *of the entire LV: Group A (19 ± 6 ms) < Group B (21 ± 8 ms) < Group C (26 ± 6 ms).

**Table 2:** Regional CMR findings between groups. Group values are presented as mean ± standard deviation

	Mean T2 (ms)		FWHM T2 (ms)		|Ecc| (%)	
	
	Septum	Free Wall	Septum	Free Wall	Septum	Free Wall
A	66 ± 10	55 ± 12*	27 ± 8	19 ± 6*	14.8 ± 0.7	14.7 ± 0.8

B	63 ± 13	53 ± 6	27 ± 10	21 ± 8	11.2 ± 1.5	12.0 ± 0.9

C	63 ± 13	66 ± 12	22 ± 7	26 ± 6	7.0 ± 0.7	7.7 ± 0.9

N1	67 ± 20	48 ± 8*	22 ± 8	20 ± 7	16.8 ± 1.0	17.6 ± 1.1

N2	59 ± 24	43 ± 9	23 ± 16	19 ± 10	-	-

Three investigators blind to the identity of the subjects independently measured the mean T2 and T2 *FWHM *of 6 subjects using the custom made software. For each subject, the mean and the standard deviation of the independent measurements were calculated. The inter-observer variability of T2 and T2 *FWHM *was assessed as the standard deviation/mean %, averaged among all the subjects. The mean percentage of standard deviation among the investigators was 3.2% for meant T2 and 3.1% for T2 *FWHM*.

## Discussion

In this study, we used the Full Width of Half Maximum *(FWHM) *of T2 distribution in LV to quantify the myocardial structural heterogeneity in DMD patients. In DMD subject groups, *FWHM *of the T2 histogram rose progressively with age and decreasing EF. Further, *FWHM *was significantly higher in those with reduced circumferential strain (|ε_cc_| ≤ 12%) (Group B, and C) than those with |ε_cc_| > 12% (Group A). The myocardial structural abnormality suggested by the observed trend is likely due to concomitant presence of micro-fibrosis (long before it becomes overt enough to be detected by the myocardial Late Gadolinium Enhancement (LGE) technique) and myocardial edema. Our results support the notion that the regional dysfunction depicted by depressed |ε_cc_| is associated with the ultrastructural myocardial cell abnormality present in DMD patients.

T2 is known to increase with intramuscular edema and inflammation that increases free water content in tissue [13] whereas fibrosis tend to decrease T2 relaxation time through increase in the collagen fractional area [14,25]. Autopsy studies have clearly shown that fibrosis and intramuscular edema co-exist in the DMD cardiac muscle [26]. Therefore the spread of T2, quantified by *FWHM *rather than the mean T2 is likely to better characterize this disease. Furthermore, LGE studies in muscular dystrophy show that distribution pattern of late enhancement is patchy and diffused unlike the well defined sub-endocardial LGE related to coronary territories found in myocardial infarction [7,24,27-29] signifying the spatial heterogeneity of the disease. The T2 *FWHM *proposed in this study quantify both spatial and pathologic heterogeneity of the DMD tissue.

Unlike the *FWHM*, the standard deviation of T2, calculated assuming that T2 is normally (Gaussian) distributed within the LV, was not significantly different between groups. This is not surprising since the histograms of T2, as seen in Figure 3, deviate significantly from that of a normal distribution. The Skewness, a measure of deviation from the normal distribution, was > 1.2 for all subjects confirmed this observation. Therefore, we conclude that the standard deviation of T2 did not accurately quantify the heterogeneity of T2 in the LV and consequently failed to distinguish DMD and normal subjects.

The mean myocardial T2 of the normal subjects (both N1 and N2 combined) in our study was 52 ± 11 ms which is same as that found by Giri et al (52 ± 3 ms) [30]. Regional analysis of T2 revealed that mean T2 was consistently lower in the lateral wall than in the septum in all groups. This may be due to the increased magnetic field inhomogeneity at the lateral wall due to the air-tissue interface. Increased B1 and B0 inhomogeneities at 3 Tesla pose significant challenges to uniform inversion of magnetization across the imaged volume affecting the accuracy of T2 calculations. As a routine practice 3D volume shimming should be employed to improve B0 homogeneity at 3 Tesla. Adiabatic radio frequency pulses, characterized by simultaneous modulation of amplitude and frequency or phase have been successful in mitigating B1 and B0 inhomogeneities [31-33]. These adiabatic pulses could be used in combination with T2 preparation schemes to generate differently weighted T2 images which then could be used to quantify T2.

### Study limitations

We used a turbo spin echo method with two acquisitions (echoes) to calculate T2. The dual echo method has been successfully used by others in cardiac and brain imaging applications in the past [13,30,34]. While it allows image acquisition to be performed during a single breath hold, it limits the accuracy of the curve fitting. However, the alternative technique of acquiring multiple echo times requires scanning to be performed during free breathing leading to volume averaging of the signal that could also affect the accuracy of the T2. Giri et al[30] recently demonstrated the superiority of T2 prepared dual echo steady state free precision (SSFP) method for measuring cardiac T2 at 1.5 Tesla. With care taken to circumvent the effects of B0 inhomogeneity, this method could be ideally suited for cardiac T2 measurement in the DMD at 3Tesla.

Although significant differences in |ε_cc_| and *FWHM *were demonstrated between young DMD patients with normal EF and older patients with reduced EF, this is a cross-sectional and not a serial study. Accordingly, repeat serial examinations would provide a more robust analysis of longitudinal |ε_cc_| and *FWHM *in this patient population. In the current study, only the midventricular slice was analyzed secondary to our experience of limited reproducibility of the basal and apical slices.

## Conclusions

The increased *FWHM *in T2 points to an underlying pathobiology that is both microscopic and widely distributed within the myocardium. Unlike gross myocardial changes that arise in the later stages of the disease, these early abnormalities are not likely to be detected by T2 weighted imaging or LGE. Thus, T2 heterogeneity quantified by *FWHM *could provide an early surrogate marker for pathobiology in DMD patients to monitor activity of disease as well as to assess efficacy of pharmacological interventions.

## Abbreviations

DMD: Duchenne Muscular Dystrophy; T2: Transverse Relaxation Time; ε_cc_: Myocardial Circumferential Strain; EF: Ejection Fraction; S_1_, S_2_: Signal Intensity of Spin Echo Images; TE_1_, TE_1_: Echo Times; *FWHM*: Full Width at Half Maximum; T2mean: mean T2 time of the Left ventricle; B0: Bore magnetic field strength; B1: Radio frequency transmit magnetic field strength.

## Competing interests

The authors declare that they have no competing interests.

## Authors' contributions

JW, KH, and WG contributed to all aspects of the manuscript's conception, design, data analysis, collection, critical revision and final approval. SH contributed to inter-observer variability analysis. WB, WM and RF contributed in data analysis, critical revision and final approval of the manuscript.
